# Is Ritonavir-Boosted Atazanavir a Risk for Cholelithiasis Compared to Other Protease Inhibitors?

**DOI:** 10.1371/journal.pone.0069845

**Published:** 2013-07-16

**Authors:** Yohei Hamada, Takeshi Nishijima, Hirokazu Komatsu, Katsuji Teruya, Hiroyuki Gatanaga, Yoshimi Kikuchi, Shinichi Oka

**Affiliations:** 1 AIDS Clinical Center, National Center for Global Health and Medicine, Tokyo, Japan; 2 Department of Community Care, Saku Central Hospital, Nagano, Japan; 3 Center for AIDS Research, Kumamoto University, Kumamoto, Japan; Temple University School of Medicine, United States of America

## Abstract

**Objective:**

To compare the incidence of complicated cholelithiasis in patients receiving ritonavir-boosted atazanavir (ATV/r)- containing antiretroviral therapy with those on other protease inhibitors (PIs).

**Design:**

We conducted a single-center retrospective cohort study of patients who started either ritonavir-boosted ATV/r- or other PIs (ritonavir-boosted fosamprenavir, unboosted fosamprenavir, lopinavir/ritonavir, and ritonavir-boosted darunavir) -containing antiretroviral therapy.

**Methods:**

The incidence of complicated cholelithiasis was determined in each group. Complicated cholelithiasis was defined as follows: 1) cholelithiasis complicated by cholecystitis, cholangitis, or pancreatitis or 2) symptomatic cholelithiasis or choledocholithiasis which required invasive procedures such as cholecystomy and endoscopic retrograde cholangiopancreatography. The effects of ATV/r were estimated by univariate and multivariate Cox hazards models as the primary exposure.

**Results:**

Complicated cholelithiasis was diagnosed in 3 patients (2.23 per 1000 person-years) in the ATV/r group (n = 466), and 3 (1.64 per 1000 person-years) in the other PIs group (n = 776), respectively. The incidence was not statistically different in the two groups by log-rank test (P = 0.702). By univariate and multivariate analysis adjusted for age and body weight, ATV/r use was not associated with cholelithiasis. (HR = 1.365; 95% CI, 0.275–6.775; p = 0.704) (adjusted HR = 1.390; 95% CI, 0.276–7.017; p = 0.690). For the 3 patients who developed cholelithiasis in the ATV/r group, the time to the diagnosis of cholelithiasis was 18, 34, and 39 months, respectively.

**Conclusion:**

In this study, the incidence of complicated cholelithiasis was low and was not different between patients on ATV/r and those on other PIs. On the contrary to ATV/r-associated nephrolithiasis, the possible risk of cholelithiasis should not preclude the use of ATV/r.

## Introduction

Ritonavir-boosted atazanavir (ATV/r) is a widely used protease inhibitor (PI) in combination with other antiretroviral drugs for patients with human immunodeficiency virus-1 (HIV) infection (URL: http://aidsinfo.nih.gov/contentfiles/lvguidelines/adultandadolescentgl.pdf) (URL: http://www.europeanaidsclinicalsociety.org/images/stories/EACS-Pdf/EacsGuidelines-v6.1-2edition.pdf). ATV/r is one of the first-line antiretroviral drugs based on its high efficacy, tolerability, favorable lipid profile, and once-daily dosing [1], [2]. However, recent studies suggested potential adverse effects associated with ATV/r, including nephrolithiasis and cholelithiasis [3], [4].

Previous studies suggested a possible causal relation between protease inhibitors and cholelithiasis [4]–[8]. Of the 20 previously reported patients with PI-associated cholelithiasis, 16 (80%) were associated with the use of ATV [4]–[8]. In one of these studies, which reported 14 patients with ATV-associated cholelithiasis, the median duration of atazanavir exposure was 42 months, suggesting that prolonged exposure to ATV is a possible risk for cholelithiasis [4]. However, there is virtually no information on the incidence of ATV/r-related cholelithiasis compared to other PIs although ATV/r is one of the most frequently prescribed PIs. Thus, we conducted a retrospective study to compare the incidence of complicated cholelithiasis in patients on ATV/r-containing antiretroviral treatment (ART) and those on other commonly used PIs [unboosted fosamprenavir (FPV), ritonavir-boosted fosamprenavir (FPV/r), lopinavir/ritonavir (LPV/r), and ritonavir-boosted darunavir (DRV/r)].

## Methods

### Ethics statement

This study was approved by the Human Research Ethics Committee of National Center for Global Health and Medicine, Tokyo. All patients included in this study provided a written informed consent for their clinical and laboratory data to be used and published for research purposes. This study has been conducted according to the principles expressed in the Declaration of Helsinki (http://www.wma.net/en/30publications/10policies/b3/17c.pdf).

### Study Subjects

This is a retrospective, single-center cohort study of patients with HIV-1 infection using the medical records at the National Center for Global Health and Medicine, Tokyo, Japan. Our facility is one of the largest clinics for patients with HIV infection in Japan with more than 2,700 registered patients. The study population was HIV infected patients, aged >17 years, who commenced treatment with ATV/r, FPV/r, FPV, LPV/r, or DRV/r-containing ART between January 1, 2004 and June 30, 2010. Both treatment-naïve and treatment-experienced patients were included. The follow-up period started at the time of commencement of ART for the first time during the study period, and ended June 30, 2011. Patients were excluded; 1) if they had started the abovementioned ART during the study period at other facilities, 2) if they were prescribed unboosted ATV. Patients with previous exposure to one of the abovementioned drugs before the present study and commenced the same drug in this study were also excluded from the analysis.

The attending physician selected the PI drug at baseline, based on the Japanese guidelines, which placed all of the abovementioned drugs as the preferred choice, at least for 3 years during the study period (http://www.haart-support.jp/guideline2011.

pdf. in Japanese). The attending physician also selected the concurrent drugs, including nucleoside reverse transcriptase inhibitors (NRTI), non-NRTI, integrase inhibitors, and CCR5 inhibitors. None of the patients received two PIs during the study period.

### Measurements

Complicated cholelithiasis was defined as follows: 1) cholelithiasis diagnosed by computed tomography or abdominal ultrasonography, together with cholecystitis, cholangitis, or pancreatitis, or 2) symptomatic cholelithiasis or choledocholithiasis requiring invasive procedures, such as cholecystomy or endoscopic retrograde cholangiopancreatography. Before the initiation of ART and until suppression of HIV-1 viral load, patients visited our clinic every month. However, after viral load suppression, the visit interval was extended up to every three months.

In this study, the primary exposure variable was ATV/r use over other PIs (FPV, FPV/r, LPV/r, and DRV/r). The potential risk factors for cholelithiasis were determined according to previous studies and collected from the medical records, together with the basic demographics [4], [9], [10]. They included age, sex, body weight, body mass index (BMI), baseline laboratory data [CD4 cell count, HIV viral load, estimated glomerular filtration rate (eGFR)], and presence or absence of other medical conditions [concurrent use of tenofovir (TDF), co-infection with hepatitis B, defined by positive hepatitis B surface antigen, and co-infection with hepatitis C, defined by positive hepatitis C viral load]. eGFR was calculated as described previously [11]. At our clinic, weight was measured on every visit whereas other variables were measured in the first visit and at least once annually. We used the data on or closest to and preceding the day of starting ART by no more than 180 days.

### Statistical analysis

Baseline characteristics were compared using the unpaired Student's *t*-test or χ^2^test (Fisher's exact test) for quantitative or qualitative variables, respectively. The time to the diagnosis of complicated cholelithiasis was calculated from the date of commencement of pre-defined PI-containing ART to the date of diagnosis of cholelithiasis. Censored cases represented those who discontinued the PIs, dropped out, were referred to other facilities, or at the end of follow-up period. The time from the start of ART to the diagnosis of cholelithiasis was analyzed by the Kaplan Meier method for patients who started ATV/r (ATV/r group) or other PIs (other PIs group), and the log-rank test was used to determine the statistical significance. The Cox proportional hazards regression analysis was used to estimate the impact of ATV/r use over other PIs on the incidence of cholelithiasis. The impact of each parameter listed above was also estimated by univariate Cox proportional hazards regression. We conducted multivariate analysis adjusted for age and body weight only, because of the small number of cases that were diagnosed with complicated cholelithiasis.

Statistical significance was defined as two-sided *p* value <0.05. We used the hazard ratio (HR) and 95% confidence interval (95%CI) to estimate the impact of each variable on cholelithiasis. All statistical analyses were performed with The Statistical Package for Social Sciences ver. 17.0 (SPSS, Chicago, IL).

## Results

**Figure 1:**
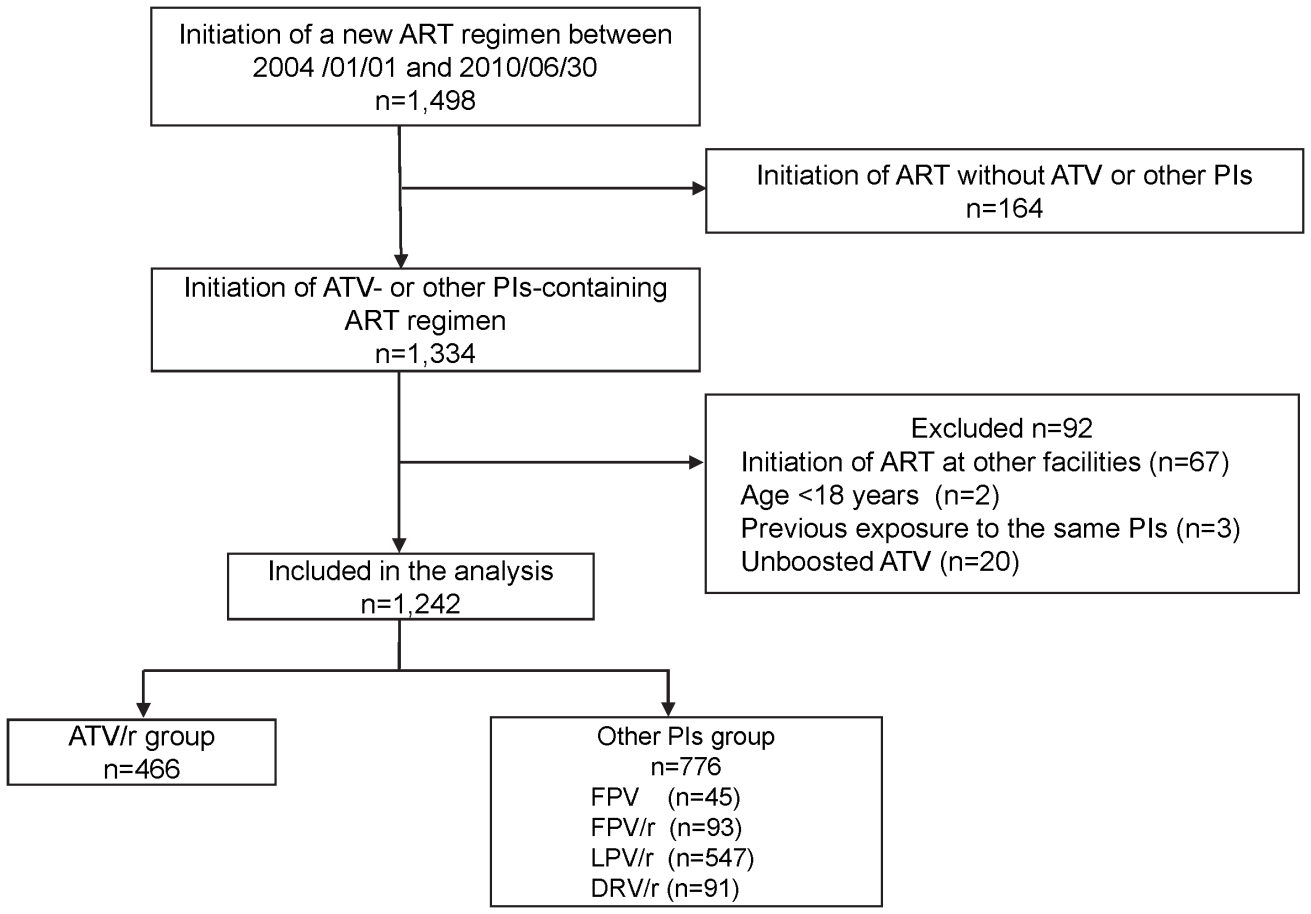
Flow diagram of patient selection. ART, antiretroviral treatment; ATV, atazanavir; PIs, protease inhibitors; LPV/r, lopinavir/ritonavir; ATV/r, ritonavir-boosted atazanavir; FPV, fosamprenavir; FPV/r, ritonavir-boosted fosamprenavir; DRV/r, ritonavir-boosted darunavir.

A total of 1,498 patients commenced or switched key drugs (PIs, non-NRTIs, or integrase inhibitor) between January 1, 2004 and June 30, 2010. Of the 1,242 patients who were included in the analysis, 466 (37.5%) started ATV/r-containing ART while 776 (62.5%) started other PIs-containing ART (Figure 1). Table 1 shows the demographics, laboratory data, and medical conditions of the study population at baseline. The majority of the study population was males, of East Asian origin, and comparatively young. The ATV/r group included significantly more patients of East Asian origin (p = 0.015) with significantly higher body weight (P<0.001), higher CD4 count (p<0.001), lower viral load (p<0.001), and lower eGFR (P = 0.012), compared with other PI groups. In contrast, patients of the other PIs group were significantly more likely to be treatment naïve (p<0.001). However, all other major background parameters were similar in the two groups.

**Table 1:** Baseline demographics and laboratory data of patients who received ATV/r- and other-PIs-containing antiretroviral therapy (n = 1,242).

	ATV/r (n = 466)	Other PIs (n = 776)	P value
Age* [SD]	39.0 [10.6]	40.0 [11.5]	0.132
Male gender (%)	434 (93.1)	714 (91.9)	0.422
Race (East Asian origin) (%)	449 (96.4)	722 (93.0)	0.015
Body weight (kg)* [SD]	65.0 [10.5]	62.1 [10.7]	<0.001
BMI (kg/m^2^)* [SD]	22.7 [3.14]	21.7 [3.25]	<0.001
CD4 count (/µl)* [SD]	304.0 [184.5]	176.2 [170.8]	<0.001
HIV viral load (log_10_/ml)* [SD]	3.58 [1.38]	4.42 [1.40]	<0.001
Treatment naïve (%)	282 (60.5)	556 (71.6)	<0.001
TDF use (%)	177 (38.0)	326 (42.0)	0.162
eGFR (ml/min/1.73 m^2^)* [SD]	117.4 [38.1]	121.7 [33.7]	0.012
Hepatitis B or C (%)	57 (12.2)	111 (14.3)	0.301

*Arithmetic mean.

ATV/r: ritonavir-boosted atazanavir, PI: protease inhibitor, SD: standard deviation, BMI: body mass index, TDF: tenofovir, eGFR: estimated glomerular filtration rate.

Cholelithiasis was diagnosed in 3 patients (0.64%) of the ATV/r group and 3 (0.39%) in the other PIs group, with an estimated incidence of cholelithiasis of 2.23 and 1.65 per 1000 person-years, respectively. The incidence was not statistically different in the two groups by log-rank test (P = 0.702). Univariate analysis showed that ATV/r use was not associated with the development of cholelithiasis (HR = 1.365; 95% CI, 0.275–6.775; p = 0.704) (Table 2). Furthermore, other variables, including gender, body weight, race, BMI, co-infection with hepatitis B or C, eGFR, CD4 count, and viral load were not associated with cholelithiasis. On the other hand, older age was associated with increased risk of cholelithiasis (per one year, HR = 1.072; 95% CI, 1.021–1.127; p = 0.006). Multivariate analysis adjusted for age and body weight indicated that ATV/r use was not associated with the development of cholelithiasis (HR = 1.390; 95% CI, 0.276–7.017; p = 0.690) (Table 2).

**Table 2:** Uni-and multi-variate analyses to estimate the risk of ATV/r use over other PIs-containing antiretroviral therapies for cholelithiasis.

	Model 1 crude (n = 1,242)			Model 2 adjusted (n = 1,203)		
	HR	95%CI	P value	HR	95%CI	P value
ATV/r use	1.365	0.275–6.775	0.702	1.390	0.276–7.017	0.689
Age per 1 year	1.072	1.021–1.127	0.006			
Male gender	0.446	0.052–3.831	0.463			
Race (East Asian origin)	0.285	0.033–2.444	0.252			
Weight per 1 kg increment	0.990	0.914–1.073	0.807			
BMI per 1 kg/m^2^ increment	0.997	0.780–1.274	0.980			
CD4 count per 10/µl increment	0.987	0.938–1.038	0.605			
HIV viral load per log10/ml increment	0.917	0.541–1.557	0.750			
Baseline eGFR 10 ml/min/1.73 m^2^ decrement	1.140	0.842–1.557	0.394			
Hepatitis B or Hepatitis C	0.040	0.000–1138.5	0.538			

Model 2 was adjusted for age and body weight.

HR: hazard ratio, CI: confidential interval, ATV/r: ritonavir-boosted atazanavir, BMI: body mass index, eGFR: estimated glomerular filtration rate.

Table 3 shows the clinical characteristics of the patients diagnosed with cholelithiasis in the present study. For the three patients of the ATV/r group, the time to the diagnosis of cholelithiasis was 18, 34, and 39 months, respectively. They were diagnosed with gallstone pancreatitis, symptomatic choledocholelithiasis, and cholecystitis, respectively, and all patients required invasive therapies. The median observation period was 31.7 months (IQR 16.0–49.7 months) for the ATV/r group and 23.0 months (IQR 10.4–42.5 months) for the other-PIs group.

**Table 3:** Clinical characteristics of patients who developed cholelithiasis.

n	Sex	Age (yrs)	BMI (kg/m^2^)	Other conditions	Protease inhibitors	Other antiretroviral agents	Duration of PI therapy (months)	Diagnosis	Invasive procedures
1	F	63	20.0	Breast cancer. hypothyroidism, hypertriglyceridemia	ATV/r	ABC, 3TC	34	Choledocholithiasis	ERCP
2	M	59	25.8	hypertriglyceridemia	ATV/r	TDF, FTC	39	Cholecystitis	PTGBD
3	M	48	29.1	hypertriglyceridemia	ATV/r	ABC, 3TC	18	Gall stone pancreatitis	ERCP
4	M	56	22.7	hypertriglyceridemia	LPV/r	ABC, 3TC	39	Cholecystitis	PTGBD
5	M	37	16.6	hypertriglyceridemia	LPV/r	ABC, 3TC	1	Choledocholithiasis	ERCP
6	M	40	19.3	hypertriglyceridemia	LPV/r	ABC, 3TC	2	Cholelithiasis	Cholecystomy

BMI: body mass index, PI: protease inhibitor, ATV/r: ritonavir-boosted atazanavir, LPV/r: lopinavir/ritonavir, ABC: abacavir, 3TC: lamivudine, ERCP: endoscopic retrograde cholangiopancreatography, PTGBD: percutaneous transhepatic gall bladder drainage.

## Discussion

To our knowledge, this is the first study that compared the incidence of complicated cholelithiasis between patients receiving ATV/r and those on other PIs. The incidence of cholelithiasis in the ATV/r group was low at 2.23 per 1000 person-years and was not statistically different from that in the other PIs groups based on uni- and multi-variate analyses.

Previous reports suggested the association between ATV/r use and cholelithiasis [4]–[6]. However, the association was not demonstrated in this cohort study of 1,242 patients. Rakotondravelo et al. reported 14 cases of PI-related cholelithiasis [4]. Although their study was not designed to calculate the incidence, the estimated incidence was 2.3 cases per 1000 person-years, which is similar to our result. This incidence is 10 times lower than that of ATV/r-associated renal stones reported in our previous study [3]. In fact, only 16 cases with ATV/r-induced cholelithiasis have been reported to date [4]–[6], compared with substantial number of ATV/r-associated renal stone reported by several groups [3], [12]–[16]. Thus, the potential risk of cholelithiasis in patients on PIs seems low compared to urolithiasis and may not be a major factor in the selection of ART.

Siveke et al. suggested that all PIs could cause cholelithiasis based on 3 cases that developed cholelithiasis while on PIs-containing ART. It is possible that PIs other than ATV/r also contribute to the development of cholelithiasis [8]. However, this cannot be confirmed at this stage and further studies are needed to address this issue.

The exact mechanism of ATV/r-induced cholelithiasis is not fully understood, although several theories have been suggested. One such theory is the precipitation of ATV in the bile with associated ATV-induced hyperbilirubinemia [4]. Another proposed mechanism relates to end-stage liver disease, which results in increased plasma ATV concentration and subsequent ATV/r-induced cholelithiasis [4]. In this study, however, we could not identify any risk factor associated with cholelithiasis.

There are several limitations to our study. First, we could not investigate asymptomatic cholelithiasis and symptomatic gallstone without complications. Thus, the risk of developing cholelithiasis associated with ATV/r might have been underestimated in the present study. Second, the prevalence of gallstones is generally lower in East Asians than in European descent and since most of the patients in this study were of East Asian origin, the effect of ATV/r might have been underestimated in our study [17]. Lastly, although prolonged exposure to ATV has been suggested as a possible etiology of ATV-induced cholelithiasis, the median observation period in our study (31.7 months) was shorter than the median latency between commencement of ATV-based therapy and the development of cholelithiasis reported in a previous study (42 months) [4]. Therefore, the short observation period in our study may have underestimated the risk of cholelithiasis. However, it remains to be determined whether ATV has a cumulative effect on the development of cholelithiasis due to the limited information available.

In conclusion, on the contrary to a substantially higher incidence of renal stones in the ATV/r group (23.7 cases per 1000 person-years) than in other PIs groups reported in the same cohort [3], the incidence of complicated cholelithiasis was low of 2.23 per 1000 person-years in the ATV/r group, and was not different between the two groups of PI-treated patients. Although the number of patients in our study might not have been large enough to show differences in the incidence of complicated cholelithiasis, the study at least suggested that the incidence of ATV/r-related cholelithiasis is low. Thus, on the contrary to ATV/r-associated nephrolithiasis, possible risk of cholelithiasis should not preclude the use of ATV/r.
